# Childhood neurodegeneration associated with a specific *UBTF* variant: a new case report and review of the literature

**DOI:** 10.1186/s12883-019-1586-x

**Published:** 2020-01-13

**Authors:** Filipa Bastos, Mathieu Quinodoz, Marie-Claude Addor, Beryl Royer-Bertrand, Heidi Fodstad, Carlo Rivolta, Claudia Poloni, Andrea Superti-Furga, Eliane Roulet-Perez, Sebastien Lebon

**Affiliations:** 1 Department woman-mother-child, Unit of Paediatric Neurology and Neurorehabilitation, Lausanne University Hospital (CHUV), Rue du Bugnon 21, 1011 Lausanne, Switzerland; 2 Great Ormond Street Hospital Institute of Child Health, University College London, 30 Guilford Steet, London, WC1N 1EH United Kingdom; 3 Department of Computational Biology, Unit of Medical Genetics, University of Lausanne, Rue du Bugnon 27, 1011 Lausanne, Switzerland; 4 Department of Medecine, Division of Genetic Medicine, Lausanne University Hospital (CHUV), Rue du Bugnon 46, 1011 Lausanne, Switzerland; 5 Department of Genetics and Genome Biology, University of Leicester, University Road, Leicester, LE1 7RH United Kingdom; 6 Department of Paediatrics, Sion Hospital, Avenue Grand-Champsec 80, 1950 Sion, Switzerland

**Keywords:** Neurodegeneration, UBTF, EEG

## Abstract

**Background:**

A new monogenic neurodegenerative disease affecting ribosomal metabolism has recently been identified in association with a monoallelic *UBTF* putative gain of function variant (NM_001076683.1:c.628G>A, hg19). Phenotype is consistent among these probands with progressive motor, cognitive, and behavioural regression in early to middle childhood.

**Case presentation:**

We report on a child with this monoallelic *UBTF* variant who presented with progressive disease including regression, episodes of subacute deterioration during febrile illnesses and a remarkable EEG pattern with a transient pattern of semi-periodic slow waves.

**Conclusions:**

This case further supports the phenotype-genotype correlation of neurodegeneration associated with *UBTF* c.628G>A. Moreover, it brings new insights into the clinical features and EEG that could possibly serve as diagnostic markers of this otherwise nonspecific phenotype.

## Background

Recently three independent teams [1–3] reported on a new monogenic neurodegenerative disease of childhood associated with a specific monoallelic *de novo* c.628G>A (p.Glu210Lys) *UBTF* variant. Together, 12 male and female patients aged 6 to 33 years old were described with a consistent phenotype of normal or close to normal early developmental milestones followed by motor and cognitive regression [1–3].

*UBTF* (*Upstream Binding Transcription Factor*, OMIM *600673) gene codes for Upstream Binding Factor (UBF), a protein that acts as a transcription factor for RNA polymerase I, essential for the generation of ribosomal RNA transcripts (rRNA) from ribosomal DNA (rDNA) in the nucleolus [1, 2]. The c.628G>A variant confers a gain of function to the protein resulting in increased expression of rDNA and rRNA, which in turn is thought to lead to scavenging of RNA binding proteins, altered RNA disposal machinery and ribosome biogenesis [1], as well as defective DNA damage repair and chromatid cohesion [1, 2, 4, 5].

We describe a further case of childhood neurodegeneration associated with this *UBTF* variant and add new insights on clinical evolution and electro-encephalographic features.

## Case presentation

**Fig. 1:**
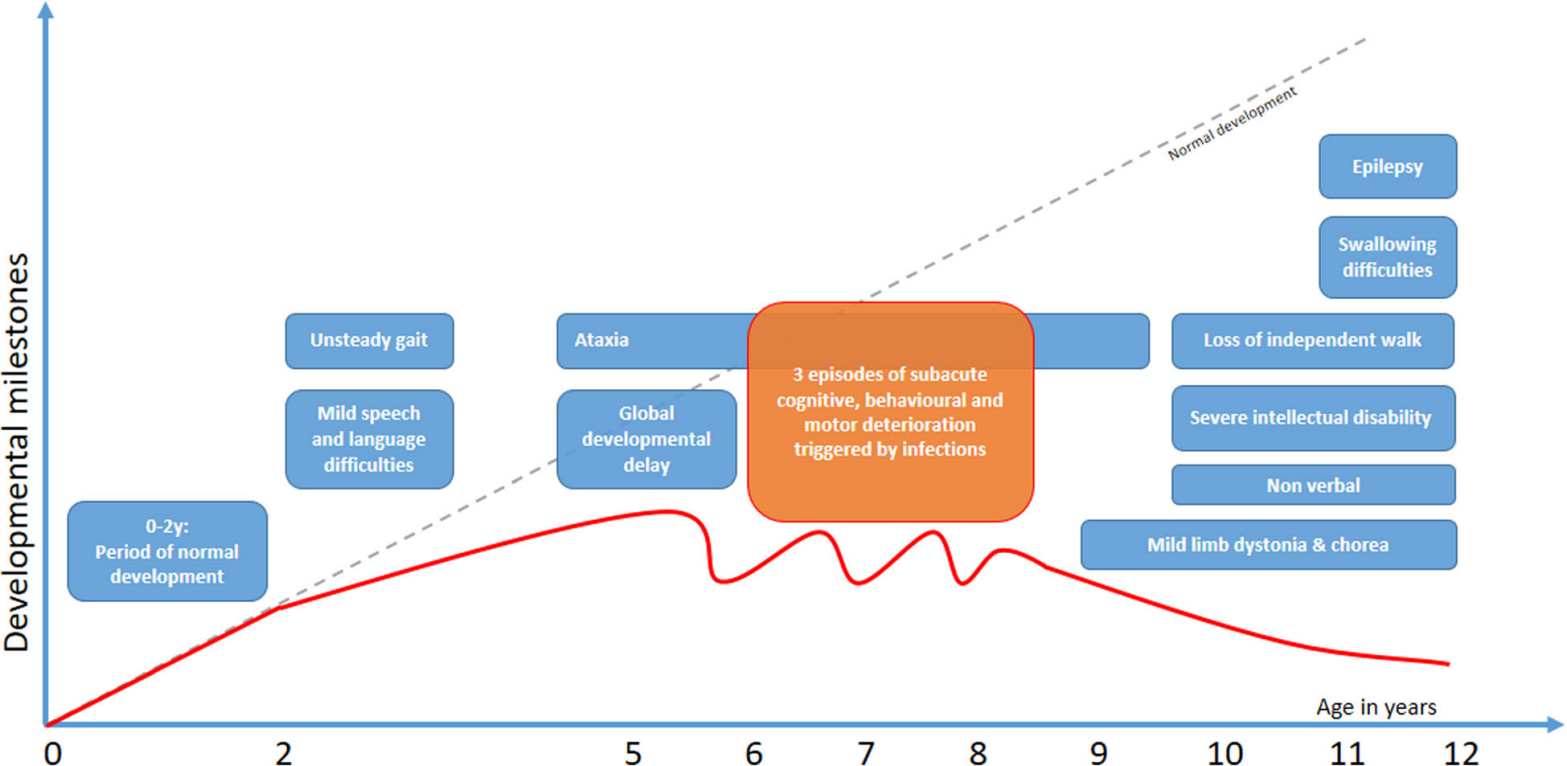
Time course of the disease. Diagram illustrating time course of disease in our patient. Dotted grey line: normal developmental trajectory; Red Line: developmental trajectory in our patient; Blue boxes: main signs and symptoms; Orange box: highlights period of subacute deterioration possibly triggered by infection

This 12-year-old boy was referred at 5 years of age for developmental delay (Fig. 1). He was the first child of unrelated sri-lankan parents; two younger siblings were healthy. His medical history was unremarkable and developmental milestones were normal up to 2 years of age, when parents noted speech and expressive language difficulties, frequent falls, and slowing of developmental progress without plateauing or loss of skills. At presentation, head circumference was at 10-25^th^ centile, height at 75-90^th^ and weight at 25^th^. On clinical examination, organomegaly or dysmorphic signs were absent. There was mild hypotonia with ataxia of limbs and gait ; cranial nerves and deep tendon reflexes (DTR) were normal. Development was globally delayed. Ten months later, following a febrile illness, the boy presented with alternating periods of agitation and apathy, loss of sphincter control, expressive and receptive language regression and worsening of the cerebellar syndrome. Three similar episodes occurred between age 6 and 8 years, all triggered by benign infectious illnesses. Thereafter, he displayed a downhill course with progressive deterioration. At the age of 11 years he developed brief epileptic behaviour arrests. At 12 years old, he has a severe intellectual disability (ID) and is nonverbal but retains a friendly behaviour. He is unable to walk unassisted and has swallowing difficulties. He has a cerebellar syndrome with mild limb dystonia and choreic movements, brisk DTR without other pyramidal signs.

**Fig. 2:**
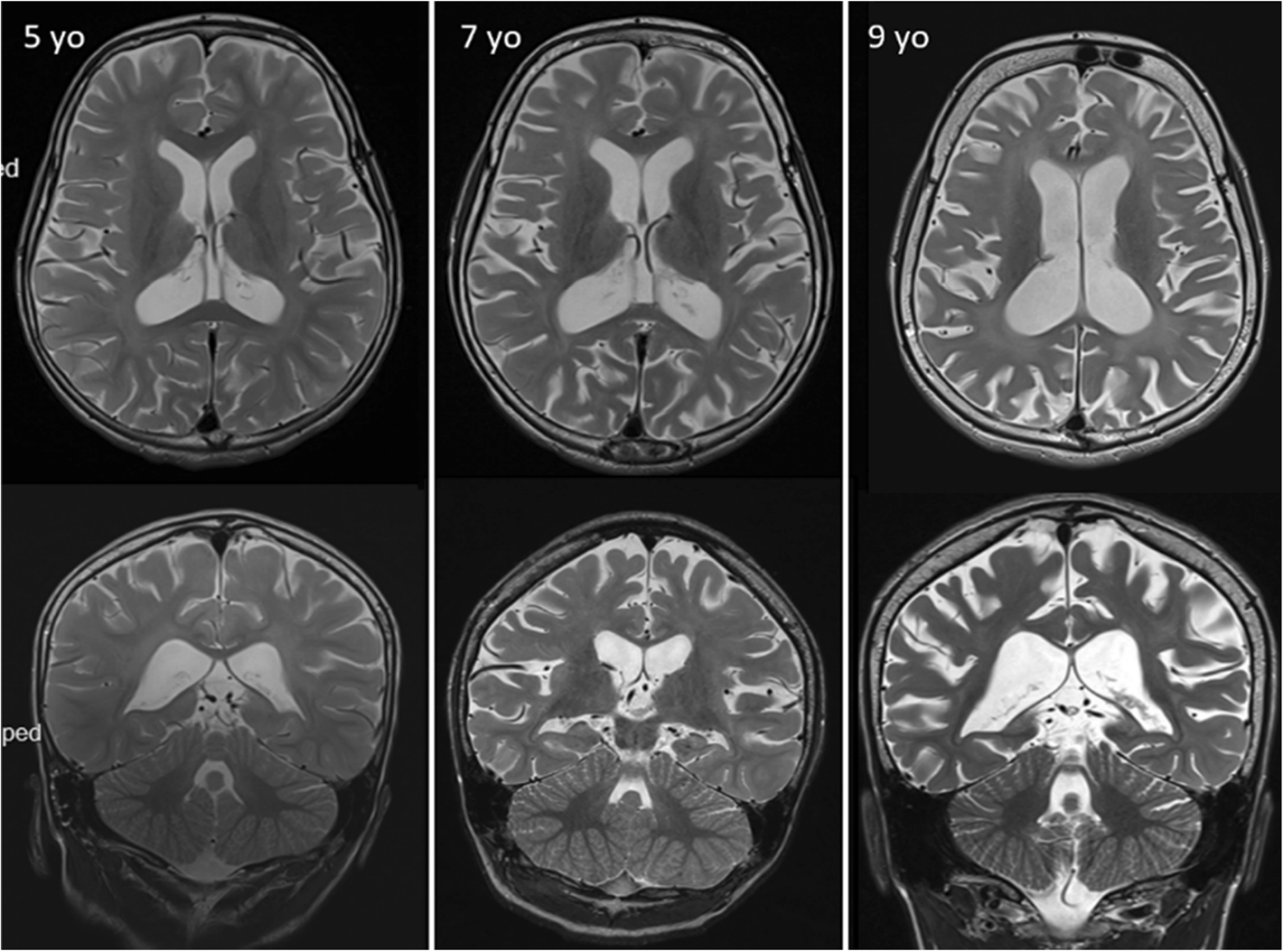
Brain MRI of the patient at different ages. Axial and coronal T2-weighted images. Supratentorial progressive cortical and subcortical atrophy with ex vacuo ventriculomegaly and diffuse deep white matter hyperintensities. Note that MRI is already abnormal at 5yo, before onset of regression. Cerebellar atrophy is only marginal. Basal ganglia, U-fibres, optic radiations, internal capsule and hippocampi were globally preserved

A first brain magnetic resonance imaging (MRI) done at 5 yo, prior to regression, showed mild white matter atrophy and periventricular hyperintensities on T2-weighted (T2W) images (Fig. 2). Follow-up MRIs done at age 6, 7 and 9 years showed progressive cortico-subcortical supratentorial atrophy, periventricular and peritrigonal deep white matter T2 hyperintensities (Fig. 2) with an increased apparent diffusion coefficient. Cerebellum appeared mildly atrophic at 9 yo.

**Fig. 3:**
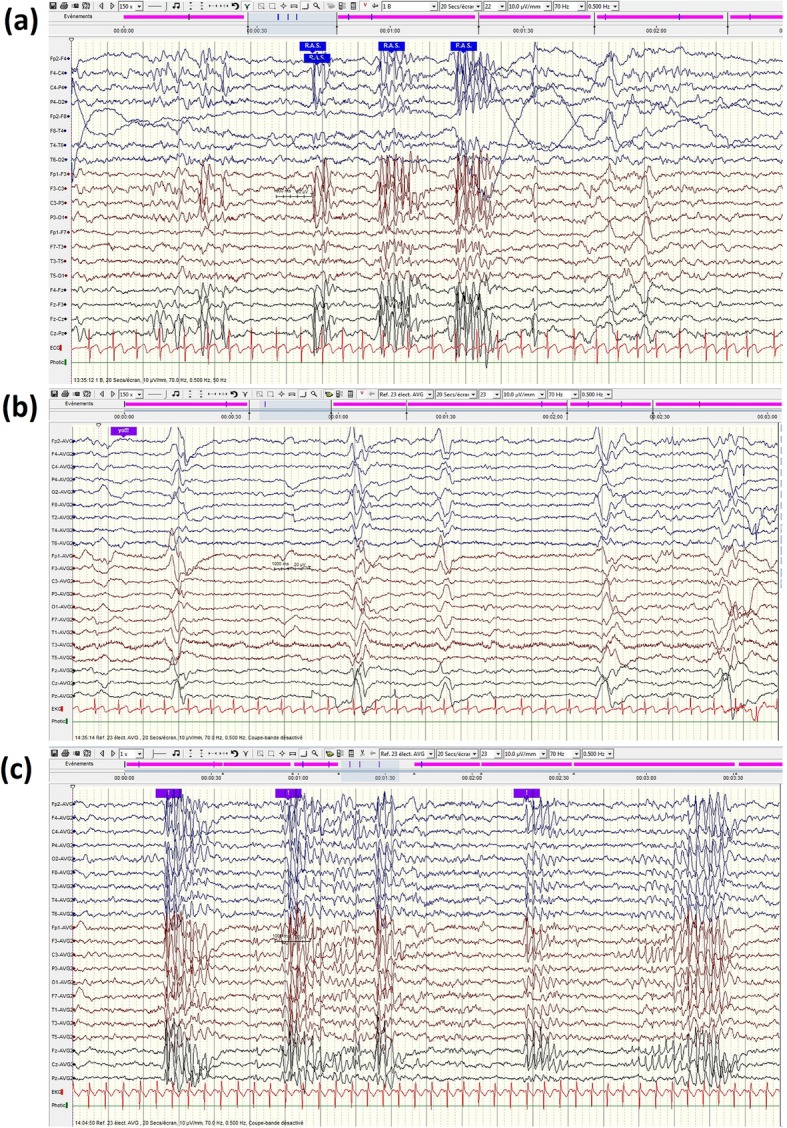
EEG of the patient at different ages. **a** 5 yo, during drowsiness bilateral bursts of frontocentral spike-wave complexes, without clinical correlate (**b**) 7yo, semi periodic delta waves without clinical manifestation; (**c**) 9yo, generalized spikes ad spike-waves complexes without clinical correlation (during sleep). Background wake activity max 7-8Hz on a,b and c

A first electroencephalogram (EEG) done at 5 yo (Fig. 3a) showed bilateral fronto-central spike and spike-wave (SW) complexes during drowsiness and sleep stage I with slightly slow background rhythm for age (7-8Hz). From 7 to 9 yo, 2 EEGs showed a pattern of semi-periodic diffuse slow delta waves complexes occurring every 2 to 5 seconds (0.2-0.3Hz) during wakefulness without clinical correlate (Fig. 3b). Sleep EEG kept on showing frequent anterior spike and SW complexes that became diffuse from 9 yo (Fig. 3c). Electroclinical seizures were recorded at 11 yo, with diffuse alpha rhythmic discharges at 11Hz during 10 seconds correlating with behavioural arrest. Photosensitivity was never elicited (minimum frequency 1 Hz). Visual and auditory evoked potentials were normal.

A first cerebral-spinal fluid (CSF) analysis during the first episode of regression at age 6 years showed 5 lymphocytes/mm^3^ and normal lactate, glucose and proteins levels. Two subsequent spinal taps at age 7 years were normal. Extensive workup including ammonium, lactate, organic acids, amino-acids, acylcarnitines profile, lysosomal enzymes activities, anti-neuronal antibodies (CSF and serum), anti-measles and anti-rubella antibodies (serum and CSF), prion and interferon signature were all negative.

At first, an Array Comparative Genomics Hybridisation (Agilent oligoNT array CGH 180K) was undertaken and did not reveal any pathogenic change. Whole Exome Sequencing was then performed on Illumina HiSeq2500 sequencer, with Agilent Sure Select XT Human All Exon V5 capture kit, after appropriate informed consent. The raw data from Whole Exome Sequencing (WES) were screened using an in-house pipeline as previously described [6] allowing for filtering of synonymous and common variants to which a panel of approximately 1300 genes known to be implicated in developmental delay and seizure disorders was applied. The original analysis failed to yield any variant that might be plausibly linked to an intellectual disability or epilepsy phenotype [7]. The data were reanalyzed six months later using an updated panel that (at that point) contained the gene *UBTF*, that had just been published in connection with an intellectual disability phenotype by Edvardson *et al* [1]. This allowed for the detection of a heterozygous c.628G>A *UBTF* variant, which was confirmed by Sanger sequencing and found to be *de novo*.

## Literature Review

The literature search yielded two case series and a case report of patients carrying the same *de novo* heterozygous c.628G>A *UBTF* variant [1–3]. Results are summarised in Table 1. Median age of onset of regression was 3 yo after a period of normal development for 10 patients. Early signs include gait ataxia, hypotonia, speech and language difficulties and behavioural and cognitive disorders. Most patients developped extrapyramidal and pyramidal signs. By early teen age, all individuals have severe ID, loss of ambulation and autonomy. No extra-central nervous system manifestations were described so far. Although EEG is abnormal in half the patients, epilepsy affected only one third of the patients, and was reported as severe in only one case [3]. Brain MRI shows predominant supratentorial atrophy and T2 deep white matter hyperintensities. Cerebellar atrophy is less remarkable but reported in the majority of cases.

**Table 1:** Phenotype of patients with the *UBTF* c.628G>A variant

	Edvardson *et al* [1] N = 7	Toro *et al* [2] N = 4	Sedlackova *et al* [3] N = 1	Present study N = 1	Total N = 13
Gender	1M/6F	2M/2F	1M	1M	5M/8F
Age at publication, yo (median, interval)	17 [8 - 23]	12 [6 – 33]	13	12	13 [6 – 33]
Age at regression, yo, (median, [interval])	3.5 [2.5 – 7]	2.7 [2 – 3]	1.3 – 2	5.8	3 [2 – 7]
DD prior to regression	3	0	Mild speech delay	Yes	5 (33%)
Microcephaly	6 acquired (1 borderline)	1 acquired (borderline)	NA	No	7/12 (58%)
Ataxia	3	4	1 (early on)	1 (early on)	9 (67%)
Extrapyramidal signs	6 (3 dystonia, 2 rigidity,1 chorea)	3 (3 dystonia, 1 chorea)	1(dystonic attacks)	1 (dystonia, chorea)	11 (85%)
Pyramidal signs	6	4	Yes	Brisk DTR	12 (92%)
Behavioural disorders	NA	4 (hyperactivity, impulsivity, ‘autistic behaviour’)	Apathy, social interaction difficulties	Apathy, agitation, friendly	6/6 (100%)
Dysarthria	NA	4	NA	Yes	5/5 (100%)
Swallowing difficulties	NA	3	Yes (11yo)	Yes (11 yo)	5/6 (83%)
Seizures/Epilepsy	3 (onset 5, 14 and 15yo)	0	Yes (multiple types) 6 yo	Yes (behavioural arrest) 11 yo	5 (38%)
Outcome	7 non verbal with profound ID, 6 non ambulatory	4 language regression, 1 non ambulatory, 1 few steps, 4 severe ID	Non verbal, severe ID, non ambulatory	Non verbal, severe ID, non ambulatory	13 non verbal or severe language impairment and severe ID (100%), 9 non ambulatory (69%), 1 limited ambulation
EEG abnormalities	4 (no details)	Abnormal background rhythms	Diffuse β activity (5yo), sharp waves (6yo), continuous bilateral FT spikes, slow background.	Yes (see text)	7 abnormal (53%)
Brain MRI	7 cerebral atrophy and T2 white matter hs, 5 cerebellar atrophy	4 cerebral atrophy, cerebellar atrophy and white matter hs	Cerebral atrophy, white matter hs	Cerebral atrophy, mild cerebellar atrophy, white matter hs	13 cerebral atrophy and white matter hs (100%), 10 cerebellar atrophy (77%)

*yo* years-old, *M* male, *F* female, *DD* developmental delay, *DTR* deep tendon reflexes, *ID* intellectual disability, *FT* frontotemporal, *hs* hypersignal, *NA* not available

## Discussion and Conclusions

After 5 years of diagnostic wandering, the finding of the specific, monoallelic *de novo* mutation c.628G>A (p.Glu210Lys) in *UBTF* allowed us to recognise a newly-described neurodegenerative disease characterized by motor, behavioural, and cognitive regression in early childhood.

Although the phenotype is nonspecific, it is in line with the previous reported cases: regression begins in pre-school years (median age 3 years) and is followed by global neurological deterioration progressing until the beginning of the second decade. Involvement is confined to the central nervous system. Speech, language and motor delay can be present prior to regression. Cognitive and behavioural degradation follows no specific pattern. Autistic traits and aggressiveness were described but absent in our case. Most patients develop cerebellar, pyramidal, extrapyramidal signs and microcephaly overtime. MRI findings are also consistent, showing cortico sub cortical atrophy with T2 hypersignal involving white matter and sometimes basal ganglia. Of note, despite a high incidence of extrapyramidal signs, basal ganglia are rarely abnormal.

Our case adds new insights on the natural history and EEG features of these patients. First, phases of neurological deterioration coincided with febrile and afebrile infectious illnesses and were followed by incomplete recovery. This finding was reported in only one other case [3] but could have been underreported. In both patients, it occurred in the first stage of the disease. Fever and infection-triggered regression is known in metabolic diseases such as mitochondrial diseases or organic acidurias [8], but also in white matter disorders such as vanishing white matter [9] for which we found no evidence. Here, fever-induced metabolic stress may have worsened the disease course because the UBF protein has a major role in rDNA transcription, and thus in ribosomal biogenesis, which is a highly energy consuming process essential for cell integrity [10]. Second, the EEG evolution was remarkable. As in others neurodegenerative disorders, there was a progressive and nonspecific slowdown of background activity. But, after the regression, we found twice, at 7 and 8 years, a peculiar pattern of generalized semi-periodic slow waves (0.2 – 0.3 Hz) without any clinical correlate. Although abnormal EEG activity was reported in about half of the reported cases, only one other case was detailed [3]. In both, interictal epileptiform activity was characterized by anterior epileptiform discharges [3]. Generalized periodic epileptiform discharges with regression, suggests the diagnosis of subacute sclerosing panencephalitis secondary to measles or rubella [11]. However, in the latter case these complexes are more regular, faster (1-3Hz), sometimes intermingled with spikes and sharp waves, can occur during sleep [11–13] and translate into myoclonus. One can wonder whether this EEG pattern may be specific of c.628 C>G *UBTF-*associated neurodegeneration and whether it relates to a particular stage of the disease. Data analysis failed to reveal other variants that might have plausibly accounted for the EEG abnormalities, either under a recessive or a *de novo* dominant model [7]. Therefore, we suggest that this might be part of the c.628 C>G *UBTF* variant phenotype.

Even though *UBTF* c.628G>A -associated childhood neurodegeneration has a consistent phenotype, no pathognomonic clinical, imaging or biological features have been identified up until now, and diagnosis was in our case only achieved by NGS. Fever- or infection-triggered episodes of regression with negative metabolic work up and a periodic EEG pattern may be useful diagnostic cues that will have to be confirmed with further case studies.

## Data Availability

The data used and analysed during the current study are available from the corresponding author on reasonable request.

## Abbreviations

CGH: Comparative Genomics Hybridisation
CSF: Cerebral-spinal fluid
DD: Developmental delay
DTR: Deep tendon reflexes
EEG: Electroencephalogram
F: Female
FT: Frontotemporal
hs: Hypersignal
Hz: Hertz
ID: Intellectual disability
M: Male
MRI: Magnetic resonance imaging
NGS: Next Generation Sequencing
NA: Not available
(r)DNA: (ribosomal) Deoxyribonucleic acid
(r)RNA: (ribosomal) Ribonucleic acid
SW: Spike-wave
T2W: T2-weighted images
UBF: Upstream binding factor
UBTF: Upstream binding transcription factor
WES: Whole Exome Sequencing
yo: Years-old
